# Immunochemical faecal occult blood tests in primary care and the risk of delay in the diagnosis of colorectal cancer

**DOI:** 10.3109/02813432.2013.850205

**Published:** 2013-12

**Authors:** Cecilia Högberg, Pontus Karling, Jörgen Rutegård, Mikael Lilja, Thomas Ljung

**Affiliations:** ^1^Department of Public Health and Clinical Medicine, Unit of Clinical Research Centre - Östersund, Umeå University, Sweden; ^2^Department of Public Health and Clinical Medicine, Medicine/Gastroenterology, Umeå University, Sweden; ^3^Department of Surgical and Perioperative Sciences, Umeå University, Sweden; ^4^Department of Health Sciences, Mid Sweden University, Sweden

**Keywords:** Colorectal neoplasms, diagnosis, general practice, occult blood, signs and symptoms, Sweden

## Abstract

**Objective:**

To evaluate the value, risks, and shortcomings of immunochemical faecal occult blood tests (iFOBTs) in the diagnosis of colorectal cancer (CRC) and adenomas with high-grade dysplasia (HGD) in patients initially presenting to primary care.

**Design:**

A retrospective population-based study.

**Setting and subjects:**

All 495 cases of CRC and adenomas with HGD diagnosed in the county of Jämtland, Sweden from 2005 to 2009.

**Results:**

Of 495 patients 323 (65%) initially presented to primary care. IFOBTs were performed in 215 of 323 (67%) patients. The sensitivity of iFOBT for CRC and adenomas with HGD was 88% (83% when patients with a history of rectal bleeding were excluded). Of 34 patients with anaemia found en passant, 10 had negative iFOBTs. Time to diagnosis was longer for patients with negative iFOBTs (p < 0.0005).

**Conclusion:**

IFOBT might be helpful in selecting which patients to refer for colonoscopy. However, iFOBT has a limited sensitivity as a diagnostic test for CRC and adenomas with HGD. Relying only on iFOBT for colonoscopy referral could delay diagnosis, especially for patients with anaemia found en passant.

Immunochemical faecal occult blood tests (iFOBTs) are used in clinical practice as diagnostic aids when colorectal cancer (CRC) is suspected. This population-based study of 323 patients with CRC and adenomas with high-grade dysplasia (HGD) in primary care shows that:

- The sensitivity of iFOBT was 83% in patients without rectal bleeding.
- Time to diagnosis was significantly longer for patients with negative iFOBTs.
- When anaemia was found en passant, iFOBTs were negative in 10 of 34 patients.

## Introduction

Worldwide, colorectal cancer (CRC) is the third most common cancer in men and the second most common in women, and in Sweden the third most common cancer in both sexes [1,2]. Colorectal adenomas with high-grade dysplasia (HGD) and a diameter over one centimetre have an increased risk of developing into cancer [3]. The majority of patients with CRC first consult primary care physicians [4]. Symptoms associated with CRC (rectal bleeding, a change in bowel habits, diarrhoea, constipation, abdominal pain) are common reasons for seeking medical advice in primary care but most people experiencing these symptoms do not have a malignant disease [5–9]. Deciding which patients to refer for further investigation is challenging, and reliable tests would help with this selection.

Faecal occult blood tests (FOBTs) are commonly used in primary care in Sweden as a point-of- care analysis to help determine whether further investigation is needed. Earlier guaiac-based tests have mostly been replaced by immunological faecal occult blood tests (iFOBTs). The latter are specific for human haemoglobin, do not require dietary instructions, and can also be qualitative or quantitative with the possibility of setting different cut-off levels. Many studies have reported on the use of guaiac-based FOBTs and iFOBTs for CRC screening [10–15]. IFOBT is now the method recommended in Europe for this purpose [16]. In spite of frequent use, there are few studies concerning FOBTs and iFOBTs and the clinical consequences of the test results in symptomatic patients in primary care [17]. One study on guaiac-based FOBTs found a limited sensitivity for CRC and that diagnosis could be delayed [18]. Another study indicated that iFOBTs were of doubtful use in clinical situations [19].

The aim of the present study was to evaluate the value, risks, and shortcomings of iFOBTs obtained in primary care in the diagnosis of CRC and adenomas with HGD. The latter were included as they are important precursors to cancer and are recorded in the Swedish Cancer Registry.

## Material and methods

In Sweden all clinicians and pathologists are obliged to report the diagnoses of CRC and adenomas with HGD to the Swedish Cancer Registry. Patients aged 18 and older residing in Jämtland, with these diagnoses reported from 2005 to 2009, were identified from the Regional Cancer Registry [20]. Jämtland is a sparsely populated county with one hospital performing all endoscopies and all surgical treatments for CRC and adenomas for its 126 600 inhabitants. There is no screening programme for CRC. Data, including free text entries, were extracted from electronic medical records in Vårdadministrativ Systemutveckling (VAS), used by all primary care centres and the hospital. All records were examined by one of the authors (CH).

When more than one CRC or adenoma with HGD was detected as a consequence of the same investigation, the clinically most severe diagnosis was chosen and considered as one case.

The start of an investigation was identified as when the patient first consulted a primary health care physician with symptoms or when the physician first initiated an investigation because of an abnormal examination or laboratory finding, leading to the diagnosis of CRC or adenoma with HGD.

Symptoms and signs were classified as gastrointestinal symptoms (a change in bowel habits, diarrhoea, constipation, and abdominal pain) with or without rectal bleeding, and/or other symptoms and signs. Rectal bleeding was defined as visible blood in the stools and/or on toilet paper.

The presence of anaemia at the start of the investigation was recorded. Anaemia was defined as a haemoglobin level < 134 g/l in men and < 117 g/l in women (the reference values used in the county's laboratories).

Patients with a visible rectal cancer or adenoma observed by the referring clinician were registered.

The date of clinical diagnosis was identified as when the CRC or adenoma was diagnosed through endoscopy, double-contrast barium enema, computed tomography, or surgery. Whether the patient had planned treatment after referral from primary care or was diagnosed after emergency admission was registered.

Data concerning all iFOBTs delivered by the patients beginning two years before the clinical diagnosis were registered. The retrospective period for symptoms and signs was also two years. Two years is the interval recommended for screening in Europe [16].

The faecal samples were analysed at the primary care centres with Actim Fecal Blood (Oy Medix Biochemica Ab, Finland), a visually read, qualitative, immunological dipstick test with a sensitivity of 50 μg haemoglobin per litre of faecal solution corresponding to 25–50 μg per gram of faeces [21]. Traditionally in Sweden a set of iFOBTs consists of three samples. Sets with one or more positive samples were regarded as positive, and sets with only negative samples as negative. Patients with first negative and at later consultations positive sets of iFOBTs were regarded as negative, as the first set was likely to have had the greatest influence on decisions about further investigation.

To calculate the sensitivity, specificity, and negative and positive predictive values we used the total number of patients who had iFOBTs analysed in the county's primary care centres from 2008 and 2009, excluding patients that were diagnosed with CRC or adenomas with HGD within two years after the test was done. The yearly number of iFOBTs analysed during this period did not differ from the previous three years. Using the statistical program SPSS version 18 (Chicago, IL, USA) we performed chi-squared tests, and when adjusting for age and sex an ANCOVA.

## Results

**Figure 1.**
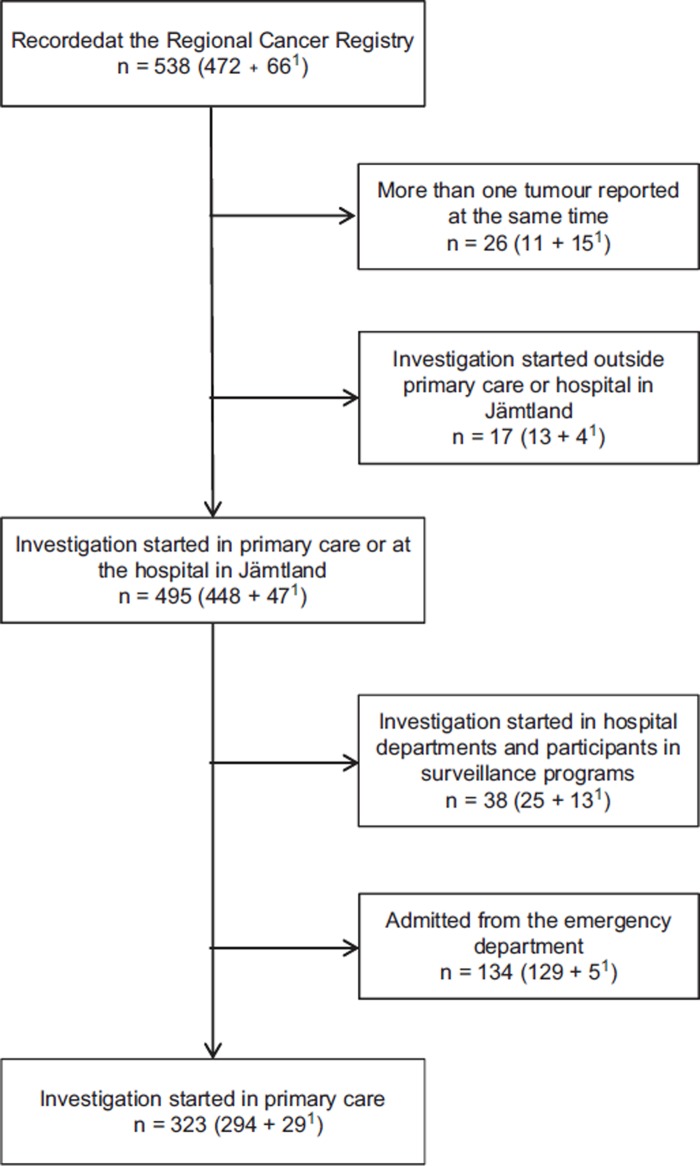
Selection of the study group, starting with 538 patients with CRC or adenomas with HGD.^1. 1^Adenomas with high-grade dysplasia (HGD).

During the study period, 294 patients with CRC and 29 patients with adenomas with HGD initially consulted primary care (Figure 1). The mean age was 71.3 (33–96) years and 48% were women.

IFOBTs were analysed in 215 (67%) patients (mean age 71.6 years, 47% women) with an average of 2.8 (1–6) samples per set of iFOBTs, in total 662 samples (Table I). In 12 patients two sets of iFOBTs, and in four patients three sets of iFOBTs were analysed. Of these 16, four had first negative and after an interval of six to 15 months positive tests.

**Table I.** Immunochemical faecal occult blood test (iFOBT) results in primary care stratified for tumour localizations in 323 patients with CRC and adenomas with HGD.

	Right-side colon n = 88 + 2^1^	Left-side colon n = 90 + 9^1^	Rectum n = 108 + 16^1^	Not specified n = 8 + 2^1^	Total n = 294 + 29^1^
No iFOBT	18 + 0^1^	24 + 2^1^	52 + 10^1^	2 + 0^1^	96 + 12^1^
Positive iFOBT	57 + 1^1^	61 + 6^1^	54 + 6^1^	3 + 2^1^	175 + 15^1^
Negative iFOBT	13 + 1^1^	5 + 1^1^	2 + 0^1^	3 + 0^1^	23 + 2^1^

Note: ^1^Adenomas with high-grade dysplasia (HGD).

Haemoglobin was analysed in all but 12 patients (Table II). Of these 12, eight had a history of rectal bleeding and all had planned treatment after referral from primary care.

**Table II.** Immunochemical faecal occult blood test (iFOBT) results stratified for the presence of anaemia at the start of investigations in primary care in 323 patients with CRC and adenomas with HGD.

	Anaemia n = 124 + 3^1^	No anaemia n = 159 + 25^1^	Unknown n = 11 + 1^1^	Total n = 294 + 29^1^
No iFOBT	23 + 1^1^	65 + 10^1^	8 + 1^1^	96 + 12^1^
Positive iFOBT	86 + 2^1^	86 + 13^1^	3 + 0^1^	175 + 15^1^
Negative iFOBT	15 + 0^1^	8 + 2^1^	0 + 0^1^	23 + 2^1^

Notes: ^1^Adenomas with high-grade dysplasia (HGD).

Anaemia was defined as haemoglobin concentration < 134 g/l in men and < 117 g/l in women.

Of the patients where iFOBTs were analysed 12% had negative results (Table III). In 34 patients where iFOBTs were performed because of anaemia found en passant, for example at annual check-ups for diabetes, no symptoms of gastrointestinal disease or anaemia were mentioned. Ten of these had negative iFOBTs, with a mean haemoglobin value of 107 g/l in cases with positive, and 103 g/l in cases with negative results.

**Table III.** Symptoms and signs at the start of investigations in primary care and results of immunochemical faecal occult blood tests (iFOBTs) in 323 patients with CRC and adenomas with HGD.

	Rectal bleeding n = 116 + 14^2^	Other than rectal bleeding, no anaemia^1^ n = 83 + 12^2^	Anaemia, no symptoms n = 33 + 1^2^	Anaemia with symptoms, rectal bleeding excluded n = 62 + 2^2^	All patients n = 294 + 29^2^
No iFOBT performed	56 + 5^2^	29 + 6^2^	0 + 0^2^	11 + 1^2^	96 + 12^2^
IFOBT performed	60 + 9^2^	54 + 6^2^	33 + 1^2^	51 + 1^2^	198 + 17^2^
of which positive iFOBT	60 + 9^2^	46 + 4^2^	23 + 1^2^	46 + 1^2^	175 + 15^2^
of which negative iFOBT	0 + 0^2^	8 + 2^2^	10 + 0^2^	5 + 0^2^	23 + 2^2^

Notes: ^1^These were gastrointestinal symptoms n = 80 + 11^2^, or only other symptoms or signs n = 3 + 1^2^.

^2^Adenomas with high-grade dysplasia (HGD).

Anaemia was defined as haemoglobin concentration < 134 g/l in men and < 117 g/l in women.

**Figure 2.**
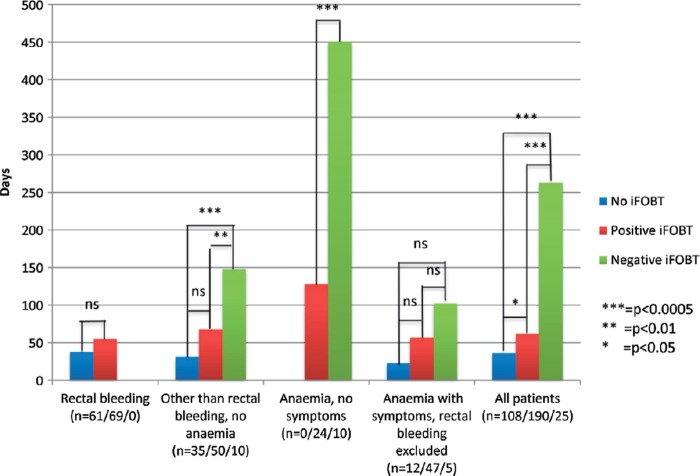
Median time from the start of the investigation to clinical diagnosis of CRC and adenomas with HGD, stratified for different symptoms and signs at the start of the investigation and the iFOBT results. Note: For the group “anaemia with symptoms, rectal bleeding excluded” and right-sided tumours, the time to diagnosis was significantly longer (p = 0.008) for those with negative (n = 4) than positive (n = 28) iFOBT at 205 (125–286) days and 85 (55–116) days, respectively. Otherwise splitting into right-sided and left-sided tumours did not affect the results.

The time from the start of the investigation to diagnosis was significantly longer for patients with negative than with positive iFOBTs (Figure 2). Adjusting for age and sex and exclusion of adenomas with HGD did not change these results. For 19% of the patients the investigation started more than 180 days before diagnosis.

Overall 27% were diagnosed after emergency admission: 26% of those with no iFOBT performed, 23% of those with positive iFOBTs, and 64% of those with negative iFOBTs.

Abnormal rectal findings were observed by the referring physician at endoscopy in 72 of the 124 patients with rectal neoplasms. In 36 (50%) of the patients with rectal neoplasms iFOBTs were performed before the endoscopy and two were negative.

The estimated sensitivity of iFOBT for CRC and adenomas with HGD was 88% with all indications for iFOBTs included. A total of 4013 patients (those with cancers and adenomas with HGD excluded) had iFOBTs performed in the county from 2008 to 2009, of which 1061 had positive sets of iFOBTs. This results in an estimated specificity of 74%, positive predictive value of 6.7%, and negative predictive value of 99.7%. Excluding patients with rectal bleeding the estimated sensitivity was 82.9%, and also excluding patients with anaemia it was 83.3%.

## Discussion

Our main finding in this population-based study of 323 patients with colorectal malignancies was that iFOBTs were often negative in patients with symptoms or anaemia. Almost one-third of the patients with anaemia found en passant had negative iFOBTs, with mean haemoglobin values equivalent for patients with negative and positive tests, indicating that less total blood loss did not explain the negative results. A longer diagnostic delay was seen in patients with negative iFOBTs compared with patients who had positive iFOBTs.

To our knowledge, this is the first study to investigate the contribution of iFOBTs performed in primary care to the diagnosis of CRC.

In this study iFOBTs were analysed in primary care in 40% of the total 538 recorded cases of CRC and adenomas with HGD, which is considerably more than the 18% of cases of CRC in a Swedish study on guaiac-based FOBTs analysed in primary care [18]. Apart from the possibility that doctors in Jämtland use iFOBTs more frequently, or that the county's residents initially consult primary care to a larger extent, the fact that all primary care centres were included in our study could contribute to this difference.

Generally, rectal bleeding is considered an alarm symptom whereby endoscopy should be performed [8,22]. Here, iFOBT before referral seems unnecessary. In patients with symptoms with low risk of CRC, iFOBT could be of value to select patients for endoscopy [23]. However, one should be aware that iFOBTs in our study were negative in approximately every sixth case of CRC without rectal bleeding. Using an iFOBT with higher sensitivity would probably give fewer false negative results, but would also most likely result in lower specificity [24]. As this study is retrospective, iFOBTs were not performed in all patients, and probably not all patients with false-positive iFOBTs were examined with colonoscopy or barium contrast enema, the sensitivity and specificity should be interpreted with caution. However, studies concerning iFOBTs used in secondary care have shown similar results [15].

Diagnostic delays in patients with negative tests have also been found in a study on guaiac-based FOBTs [18]. It seems probable that doctors use iFOBTs as a means for deciding on further investigation, and are misled by negative results. In our study, the diagnostic delays were most evident for patients with anaemia found en passant, and for symptomatic patients without rectal bleeding or anaemia. These subgroups, where a reliable test in clinical practice would be of greatest value, had a sensitivity of 71% and 83%, respectively. As mentioned above, iFOBT can be useful in primary care, but negative results cannot be entirely trusted.

Of the patients whose investigation started in primary care, 27% had an emergency admission, which is similar to findings in other studies [25,26]. We found that the group with negative iFOBTs had a larger proportion (64%) of emergency admissions, indicating that a missed diagnosis in many cases resulted in acute illness.

Our study has several weaknesses. It is retrospective and iFOBTs were not analysed in all patients. In the group where no iFOBTs were performed, it is probable that a higher percentage had more evident symptoms and was referred without further investigation. The symptoms retrieved from the patients’ records were probably not all the symptoms mentioned. On the other hand it is likely that doctors registered what they considered most important for their decision-making. Validation of data extraction was not done, but all records were analysed by the same person.

The study also has its strengths. The population was well defined, and the coverage in the Swedish Cancer Registry is almost complete [27]. All patient records are kept in the same computer system, and were examined by one person. IFOBTs were performed in two-thirds of the patients, the same method was used in all analyses, and the date of diagnosis is exact.

In conclusion, iFOBT might be helpful in selecting patients to be investigated with endoscopy. However, one should be aware of the limited sensitivity of iFOBT as a diagnostic test, for CRC and adenomas with HGD, especially in patients with anaemia found en passant. IFOBT in combination with certain symptoms and other laboratory tests could, it is hoped, improve the diagnosis of CRC. Further research concerning this is needed.
